# Discrimination of Ignitable Liquid Residues in Burned Petroleum-Derived Substrates by Using HS-MS eNose and Chemometrics

**DOI:** 10.3390/s21030801

**Published:** 2021-01-26

**Authors:** Barbara Falatová, Marta Ferreiro-González, José Luis P. Calle, José Ángel Álvarez, Miguel Palma

**Affiliations:** 1 Department of Fire Protection, Faculty of Wood Sciences and Technology, Technical University in Zvolen, ul. T. G. Masaryka 2117/24, 960 53 Zvolen, Slovakia; xfalatova@is.tuzvo.sk; 2 Department of Analytical Chemistry, Faculty of Sciences, IVAGRO, ceiA3, University of Cadiz, 11510 Puerto Real, Spain; joseluis.perezcalle@uca.es (J.L.P.C.); miguel.palma@uca.es (M.P.); 3 Department of Physical Chemistry, Faculty of Sciences, Institute of Biomolecules (INBIO), University of Cadiz, 11510 Puerto Real, Spain; joseangel.alvarez@uca.es

**Keywords:** hierarchical cluster analysis, linear discriminant analysis, headspace–mass spectroscopy electronic nose, fire debris, total ion mass spectrum, ignitable liquid residues, interferences

## Abstract

Interpretation of data from fire debris is considered as one of the most challenging steps in fire investigation. Forensic analysts are tasked to identify the presence or absence of ignitable liquid residues (ILRs) which may indicate whether a fire was started deliberately. So far, data analysis is subjected to human interpretation following the American Society for Testing and Materials’ guidelines (ASTM E1618) based on gas chromatography–mass spectrometry data. However, different factors such as interfering pyrolysis compounds may hinder the interpretation of data. Some substrates release compounds that are in the range of common ignitable liquids, which interferes with accurate determination of ILRs. The aim of the current research is to investigate whether headspace–mass spectroscopy electronic nose (HS-MS eNose) combined with pattern recognition can be used to classify different ILRs from fire debris samples that contain a complex matrix (petroleum-based substrates or synthetic fibers carpet) that can strongly interfere with their identification. Six different substrates—four petroleum-derived substrates (vinyl, linoleum, polyester, and polyamide carpet), as well as two different materials for comparison purposes (cotton and cork) were used to investigate background interferences. Gasoline, diesel, ethanol, and charcoal starter with kerosene were used as ignitable liquids. In addition, fire debris samples were taken after different elapsed times. A total of 360 fire debris samples were analyzed. The obtained total ion mass spectrum was combined with unsupervised exploratory techniques such as hierarchical cluster analysis (HCA) as well as supervised linear discriminant analysis (LDA). The results from HCA show a strong tendency to group the samples according to the ILs and substrate used, and LDA allowed for a full identification and discrimination of every ILR regardless of the substrate.

## 1. Introduction

Proper identification of ignitable liquid residues (ILRs) in fire debris is complex in itself since the remaining samples after a fire contain substrate background compounds as well as other products from combustion and pyrolysis processes. For this reason, one of the challenges for forensic analysts consists of isolating ILR’s target compounds from either background or pyrolysis compounds that may interfere with the analysis and obstruct proper identification of the target compounds [1,2]. Therefore, a preconcentration step prior to the analysis, which is normally based on gas chromatography–mass spectrometry (GC-MS), is usually carried out. The most common method for the separation of ILRs in fire debris samples is based on headspace analysis using activated carbon strips as sorbents, as described in the American Society for Testing and Materials (ASTM) E1412 [3]. Other alternatives for the preconcentration, also for headspace analysis of the samples, include solid phase microextraction [4], statistic headspace adsorption with tenax [5] or headspace sorptive extraction [6]. The analysis of the static or dynamic headspace without any adsorbent has also been applied [7,8].

Once the analysis is performed, data interpretation is a complex endeavor that requires specific skills, expert knowledge, and specific technology to be carried out. The gathered data, and its analysis should be considered objectively, without any biased expectations, nor any prejudices or preconceptions. According to the classification scheme provided by the ASTM E1618-14 [9] there are eight classes of ignitable liquids (ILs). The ASTM E1416 deals with the identification of ILRs in fire debris extracts by means of gas GC-MS. The standard also describes three methods for data analysis and IL classification, i.e., chromatographic pattern recognition, extracted ion profiling, and target compound analysis. Chromatographic pattern recognition is performed by comparing chromatograms to a reference database; extracted ion profiling is based on selected ions/group of ions (*m*/*z*) to visualize distinctive patterns of specific IL classes; and target compound analysis allows the identification of target compounds in ASTM gasoline and distillate classes. Thanks to target compound chromatograms (TCC) analysts can visualize even really low concentrations of ILRs. However, this method does not establish a threshold for the presence of an ILR in a fire debris sample. Additionally, it does not establish ILRs’ intensity limits required to determine their presence or absence [9]. Thresholds are individually set and can be biased depending on the analyst [10]. According to a U.S. national study [11], increased reliance on forensic sciences statistical methods is recommended. Over the past 30 years there has been a significant increase in applications of chemometric methods to the analysis of ILs and fire debris. Apart from simple classification of ILS by ASTM classes and small data sets, chemometrics have been applied to extensive data sets and create thousand examples for training [12]. Despite the fact that the most of data came from GC-MS, alternative analytical techniques such as Raman spectroscopy, headspace–mass spectroscopy electronic nose (HS-MS) or others were also proven useful. Monfreda and Gregori [13] investigated gasoline samples by solid-phase microextraction (SPME) and GC-MS. By applying principal component analysis (PCA) as well as discriminant analysis (DA) to a data matrix obtained by target compound chromatogram, they were able to discriminate samples by brand. Pearson product moment correlation (PPMC) and PCA were applied to total ion chromatogram (TIC) and extracted ion chromatograms (EICs) of the chosen ions from diesel samples. Thirteen different diesel brands were clustered based on common trait [14]. Mat-Desa et al., by employing PCA, hierarchical cluster analysis (HCA), and a self-organizing feature map (SOFM) and artificial neural network (ANN) to chromatographic data, successfully classified neat lighter fuels [15] and medium petroleum distillate (MPD) products [16] (samples) and linked weathered samples (under controlled conditions) with their unevaporated counterparts. Zorzetti et al. [17] by means of GC × GC—FID (flame ionization detector) and by applying a variety of chemometric models, such as partial least squares—discriminant analysis (PLS-DA), nonlinear PLS (PolyPLS), and locally weighted regression (LWR) were able to predict how long a sample had been exposed to evaporative weathering. Baerncopf et al. [18] applied PPMC and PCA to simulated fire debris samples analyzed by HS-GC-MS. Six different ILs were used together with nylon carpet as substrate. The results indicated that chemometric tools link each ILR to its neat IL, even in the presence of matrix interferences. Sinkov et al. [19] in 2014 used a chromatographic alignment strategy based on a ladder that consisted of perdeuterated n-alkanes. By PLS-DA and soft independent modelling of class analogies (SIMCA) applied to specific arson, a 100% of correct classification of samples based on gasoline content was achieved. It has been previously reported [20] that projected difference resolutions (PDRs) is a powerful chemometric tool to compare different analysis methods in terms of pattern recognition.

In 2008, Sigman et al. [21] pointed out that total ion spectrum (TIS) provides an alternative approach to data analysis. TIS is identical to an average mass spectrum (MS) that would cover the complete chromatographic range. In this sense, Waddell et al. [22] stated, that one of the challenges of fire debris classification by different laboratories may lie in the comparison of chromatographic data, specifically TICs. Waddell et al. [22,23,24,25] applied various chemometric tools to the total ion spectrum (TIS) of the samples from the ignitable liquids and reference collection database (https://ncfs.ucf.edu/databases/ilrc-2/) [26] in order to achieve correct classification rates in fire debris analysis. A recent study demonstrated the application of likelihood ratios and optimal decision thresholds based on PLS-DA by using the TIS. In this study, the authors emphasized the connection between quantified strength of the evidence and categorical decisions based on a defined operational decision point on the ROC curve [27]. 

During recent years, several research groups have proposed some alternative methodologies. Aliaño-González et al. have used the ion mobility spectrum obtained from the headspace for the identification of IL in fire debris [28]. In similar way, P. Calle et al. have recently used the ion mobility spectrum in combination with linear discriminant analysis (LDA) to characterize biodegraded ILs [29]. 

Headspace mass spectrometry electronic nose (HS-MS eNose) combined with chemometric tools have also been successfully applied to fire debris analysis. This technique runs the analysis using samples from the static headspace with a mass detector system, but without any chromatographic separation. Thus, it presents several advantages when compared to the two other methods previously discussed. It provides total ion mass spectrum (TIS) as an overall fingerprint of volatile profile. This is the same as in TIS, but it is quick and does not require any solvent or adsorbent. HS-MS eNose employs a quadrupole mass spectrometer in which each fragment ion (*m*/*z* ratio) of the detector acts as a “sensor”. Ion abundance varies with the sensor signal [30]. Moreover, the “mass sensor” provides chemical information from each sample. As above mentioned, multivariate statistical analysis combined with HS-MS eNose [31,32,33,34] have been successfully applied in different fields including fire debris analysis [7,8]. Ferreiro-González et al. have successfully applied HS-MS eNose to thermal desorption of ILRs from carbon strips as an alternative to CS_2_ as solvent [35]. HS-MS eNose was also applied to discriminate different gasoline samples as well as petroleum-based products in water samples [36,37]. In a previous study the effect of weathering on neat gasoline [38] as well as the effect of fire suppression agents on the interpretation of the results from fire debris analyses were assessed [39]. All of these phenomena, as well as the use of interfering substrates, can alter typical chemical fingerprints and, therefore, lead to a wrong interpretation of the data. For this reason, the aim of this study is to develop a robust model for the identification of ILRs in fire debris, even in the presence of complex matrices that may comprise similar products to some typical ILs. Such matrices may contain products that are similar to ILs typically used as fire accelerants. Therefore, petroleum-derived substrates such as vinyl, linoleum, polyester, and polyamide carpet together with other matrices were used as the support material, and gasoline, diesel, charcoal starter with kerosene, and ethanol (petroleum-based products) as the IL to start the fire. TIS in combination with HCA and LDA was used for discrimination purposes.

## 2. Materials and Methods

### 2.1. Fire Debris Preparation

Six different petroleum-derived substrates—four flooring materials: vinyl flooring (43% ethylene and 57% chlorine), linoleum flooring (limestone, wood powder, and linseed oil), polyester carpet (100% polyester), and polyamide carpet (100% polyamide), as well as two different materials for comparison purposes (100% cotton sheet and natural cork) were used in this study. Substrates were obtained from local stores in Cadiz, Spain. Four different ILs were used for burning. Gasoline (95 Research Octane Number, lead-free) was purchased from a gas station in Alcalá de Henares, Madrid (Spain). Diesel (cetane index > 45) was purchased from another gas station in Jerez de la Frontera, Spain. Ethanol absolute (99.8%) from Panreac (Barcelona, Spain) and charcoal starter with kerosene (naphtha (petroleum), hydrotreated heavy, kerosene,) were obtained from local stores. Fire debris preparation followed the modified procedure Destructive Distillation Method for Burning [40]. One piece of substrate (5 × 5 cm) was replaced by six small pieces (1 × 4 cm) and placed on the bottom of a metal can. 0.5 mL of each ignitable liquid (IL) was applied onto the substrates; respectively, gasoline, diesel, ethanol, and charcoal starter were used. Subsequently, the can with a punched lit was placed on a propane torch. When smoke started to visibly come out through the holes in the lid, the can was left for two minutes on the burner. Then it was removed from the burner and let to cool down. The punched lid was replaced by a solid lid when the can cooled down. Samples of the fire debris were taken after 10 min, 1, 6, 12, 24, and 48 h. These times were selected since in a real situation the fire debris samples are taken after the fire is extinguished and the scene conditions are safe what it usually takes hours or even days. The fire debris samples were labelled as follows: FD for fire debris (FD) and then V for vinyl, LIN for linoleum, N for polyamide carpet, PO for polyester carpet, CS for cotton sheet, CO for cork and finally, an indication of the elapsed time (0 h, 1 h–48 h). Ignitable liquids were identified as follows: GAS for gasoline, DIE for diesel, ETH for ethanol and KER for charcoal starter with kerosene. All the possible combinations of substrate and ILR were prepared. Then, the burned samples were identified by their substrate code followed by a liquid code and the elapsed sampling time. For instance: FD_V_1H for burned vinyl substrate without IL when sampling was performed 1 h after the burning. FD_N_GAS_6H for burned polyamide carpet with gasoline when sampling was performed 6 h after the burning. After combustion, all of the samples were kept at a controlled room temperature (25 °C). After sampling, the fire debris samples were placed directly into vials and analyzed by HS-MS eNose. A total of 360 fire debris samples were obtained.

### 2.2. HS-MS eNose Spectra Acquisition

All of the fire samples were analyzed by an Alpha Moss electronic nose (Toulouse, France) based on headspace (HS 100 static headspace autosampler) and mass spectrometer (Kronos quadrupole mass spectrometer). The experimental conditions used for the analysis (incubation temperature 115 °C and incubation time 10 min) were previously optimized and described in another study [8]. In order to avoid cross-contamination, after each injection, the gas syringe was flushed down with nitrogen and between fire debris samples a blank was also analysed. Each analysis lasted 15 min.

Residual Gas Analysis software and Alpha Soft 7.01 software package (Alpha Moss, Toulouse, France) was used for instrument control.

### 2.3. Data Analysis

Total ion mass spectra (TIS) in the range of 45–200 mass-to-charge ratios (*m*/*z*) from fire debris samples were obtained and set into a data matrix D*_mx_*_n_, where *m* is the number of fire debris samples and *n* is the number of *m*/*z* intensities in the spectral range. Each *m*/*z* intensity was considered as independent variables. All of the TIS were standardized by assigning one unit to the maximum intensity.

Hierarchical cluster analysis and graphics were produced using R-Studio software (RStudio Team (2020), Boston, MA, USA), and supervised linear discriminant analyses were performed by means of IBM SPSS Statistics 22 software (Armonk, NY, USA).

For each LDA, stepwise method was chosen to select the most significant variables (*m*/*z*). In addition, Wilke’ Lambda value was used as a criterion to introduce or eliminate variables and the input *F*-value was 3.84 and the output *F*-value was 2.71.

## 3. Results and Discussion

### 3.1. Exploratory Study

**Figure 1:**
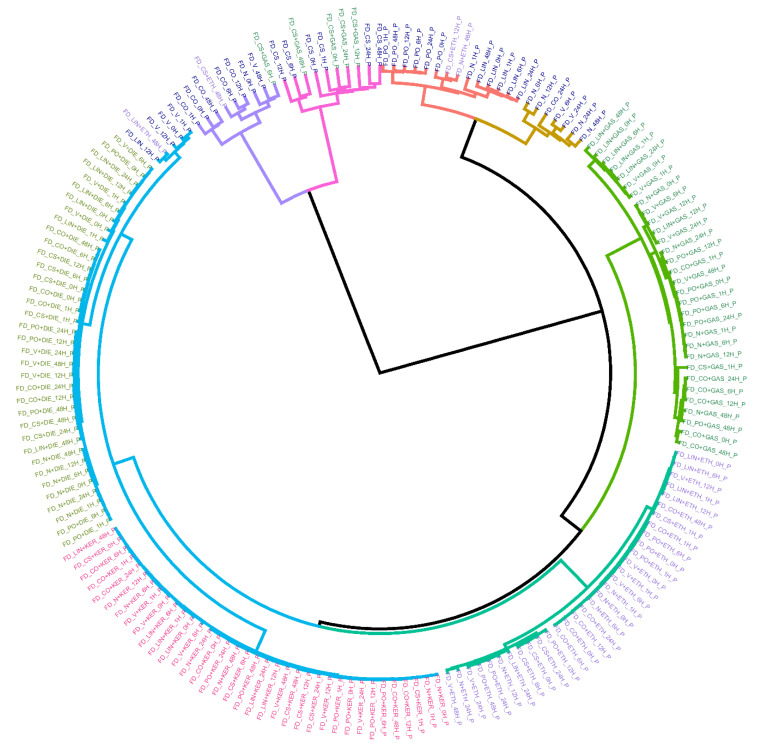
Dendrogram resulting from the hierarchical cluster analysis (HCA) analysis using average values of the replicas. Ward´s method with Manhattan distances were used. Samples are colored according to the type of IL use for burning: DIE (light green), GAS (dark green), KER (pink), and ETH (purple) and substrate burned without IL (dark blue).

First, the tendency of the samples to cluster according to the presence/absence of IL, the type of IL or substrate as well as the sampling time was checked. For this purpose, the whole set of fire debris samples (*n* = 360) was analyzed by the headspace mass spectrometry electronic nose (HS-MS eNose). The total ion mass spectrum (TIS) of each sample was obtained and normalized by assigning one unit to its maximum intensity. First, HCA (hierarchical cluster analysis) was carried out as exploratory technique. For this analysis, Ward’s method with Manhattan distance were used. The results are shown in a dendrogram (Figure 1). Since there are a high number of samples, the dendrogram is displayed in a circular way and, a reduced data matrix corresponding to the mean values of the replicas was used for an easier interpretation (D_180×156_). 

As can be seen, samples with the same type of ILs are very close together, as well as the same substrates. Therefore, there is a strong trend of the samples to be classified based on the ILs used and on the type of substrate. Regarding the samples burned with KER and DIE, it is observed that these samples are included in different subclusters but joined at a short distance giving rise to a larger cluster (cluster colored in blue). This is most likely due to the similar chemical composition of both ILs. In fact, KER and DIE belong to the same category according to ATSM [9]. At a higher distance, a separated cluster (cluster colored in dark green) containing samples burned with ETH is joined to the previous blue cluster. Only four samples burned with ETH are misclassified and included in groups containing substrates burned without ILs. These “misclassified” samples were taken 12–48 h after the burning, so the misclassification can be due to the high volatility of ethanol. This is the only case in this study where the sampling time seems to influence the clustering. Focusing on samples burned with gasoline most of them are included in the same cluster (cluster colored in light green) except for five samples. It is remarkable that these five misclassifications belong to the same substrate, CS (cotton sheet) and, that these samples are included together with substrates burned without IL. This is maybe due to the different porosity of CS in comparison to the other substrates. In addition, cotton is a non-interfering substrate, so it produces very few signals in comparison to the petroleum-derived substrates. It is also important to highlight, that the samples burned with the same liquid tend to be classified within each cluster in different groups according to the type of substrate.

Finally, the samples burned without ILs are included in different main clusters according to the type of substrate. On the one hand, non-petroleum derived substrates (CO and CS) are grouped in a main separated cluster divided into two clusters (clusters colored in purple and in pink) far from the rest of the substrates. On the other hand, most of the petroleum-derived substrate samples (cluster colored in red and in mustard) are grouped in the same main cluster that include samples burned with IL. In particular, they are closer to samples burned with gasoline. These results indicate that TIS from these substrates have common signals with TIS from ILs and therefore, they could interfere with the correct classification. Hence, it can be stated that there is a strong clustering trend depending on both the type of IL and the substrate used. The strong influence of both, ILs and substrates hide the influence of signal related to sampling time in most of the cases. Only, sampling time seems to be influential in the case of samples burned with ethanol and samples after 12 or 48 h. Based on exploratory analysis, the sampling times evaluated in this work cannot be considered an important factor for the identification of the ILs. Nevertheless, using all the signals derived from the mass spectrum a full separation based on the type of ILs is not possible using HCA. For this reason, a supervised technique such as linear discriminant analysis (LDA) was used.

### 3.2. Detection of the Presence/Absence of ILR

Based on the high influence of the substrate showed by HCA, the next step was to determine if any ignitable liquid (IL) had been used or not to start the fire regardless the substrate. To do so, a chemometric method—supervised pattern recognition linear discriminant analysis (LDA)—was applied to the whole dataset D_360×156_ in order to develop an approach that was capable of detecting the presence of any ignitable liquid residue (ILR) regardless neither of the substrate used nor of the sampling time. Then, two classes were established a priori according to the presence/absence of ILR (ILR/no ILR), that would allow to develop a robust model. For that purpose, the original dataset (*n* = 360 samples) was divided in two datasets. In particular, 70% of the original dataset was randomly (but guaranteeing representativeness of all groups) put aside (*n* = 254 samples) as a “training set” to develop a model, while the remaining data (*n* = 106 samples) was used as “validation set”. A stepwise method was performed with the LDA in order to search for those m/z signals that were more relevant to discriminate the absence or presence of ILR in the burned samples. One hundred percent of the samples were correctly classified in both the training set and as well as in the test set. Table 1 includes the coefficients of the resulting Fisher´s linear discrimination functions.

**Figure 2:**
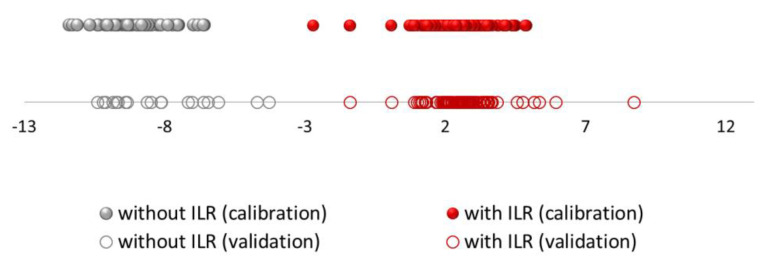
Discriminant scores obtained by LDA for all the burned samples using headspace–mass spectroscopy electronic nose (HS-MS) data (the red colour corresponds to samples with ignitable liquid residues (ILR) and the grey colour without ILR); The dots represent the samples in the training set and the circles represent the samples in the validation set.

Figure 2 displays the discriminant scores obtained from LDA for both sets of samples (training and validation sets). The magnitude of the actual effect of predictors and outcome was 0.954. 

According to the *p*-value of the model (*p* < 0.05), groups of predicted variables will make predictions that are statistically significant in their accuracy, so the resulting model is very stable and reliable. Within each group, a fairly homogenous grouping can be observed. As it can be seen, the group of samples without ILR (represented in grey colour) is more homogeneous. However, it can be seen how the group of samples with ILRs (represented in red colour) are more widely distributed since the group is more heterogeneous probably due to the type of IL used and the amount of ILR in the sample.

### 3.3. Discriminating the Different Types of ILRs

**Figure 3:**
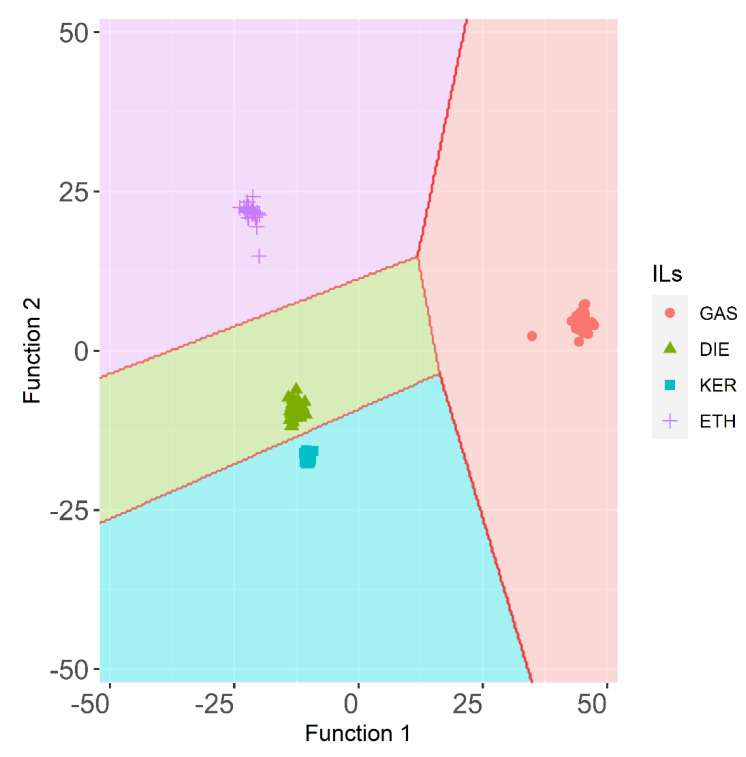
Territorial map for the ILR samples (training set) obtained by LDA using HS-MS eNose data (*n* = 202).

Therefore, after successfully determining the presence/absence of IL in fire debris samples, the last step consisted of identifying specific ILRs in them. To do so, the samples that had been burned without any IL were discarded for any further studies. A new stepwise method including supervised LDA was then applied to a D_288×156_ data matrix. Seventy percent of the fire debris samples (*n* = 202) were randomly (but guaranteeing representativeness of all groups) used to develop the model. In this case, four groups of fire debris samples corresponding to each ignitable liquid were established a priori: GAS, DIE, KER, and ETH. Three canonical discriminant functions were used to explain the result of the analysis (Function 1, Function 2, and Function 3), with the following percentages of variance: sequentially 86.7%, 11.1%, and 2.2%. LDA allowed for a full discrimination (100% of the samples were correctly classified in both, training set, and the validation set) thanks to a very stable prediction model (*p* < 0.05). The territorial map obtained from this LDA is represented in Figure 3.

The territorial map plots give the location of cases based on their discriminant scores. For GAS samples longer average distance among the samples than for to other samples was found. ETH samples also show higher average distance than DIE or KER samples. Fire debris samples that had been burned with these two ILs show short distances between their centroids. Additionally, the KER group is the most homogeneous one, which means that, regardless of the substrates or the sampling time, the results are similar to those obtained for samples burned with DIE. Therefore, samples containing DIE or KER are quite improbable to be mistaken for other ILRs.

**Figure 4:**
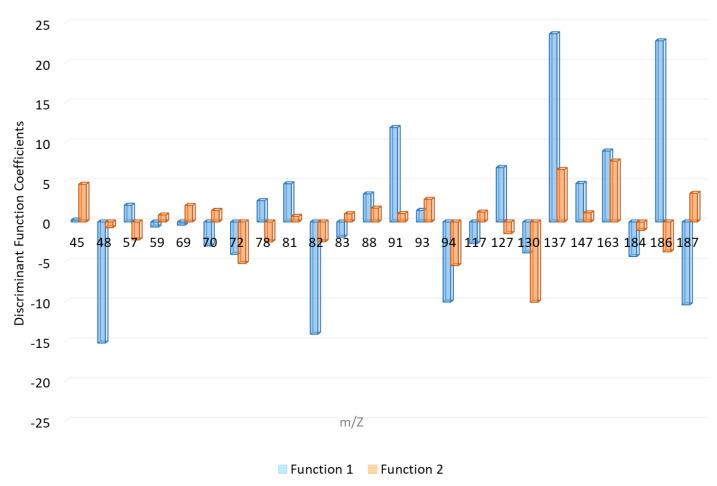
Standardized canonical discrimination coefficients.

The canonical discriminant functions enable full discrimination of ILRs from fire debris samples. As can be seen gasoline samples are concentrated on the positive end of function 1, consistent with the location of their group means (centroids). Whereas, function 1 separates GAS samples, it does not allow to fully separate the remaining samples. The function 2 provides full separation between samples burned with ETH, DIE, and KER. The standardized canonical discriminant function coefficients of the function 1 and 2 are shown in Figure 4. As can be observed the highest positive values in function 1 are due to the signals *m*/*z* 81, *m*/*z* 91, *m*/*z* 127, *m*/*z* 137, *m*/*z* 147, *m*/*z* 163, and *m*/*z* 186 while the negative values are due to *m*/*z* 48, *m*/*z* 82, *m*/*z* 137, *m*/*z* 94, and *m*/*z* 187. 

Based on the related chemical compositions of diesel and the charcoal starter that contains kerosene, the samples burned with one of these two ILs obtained very similar negative values from both discriminant functions. The samples burned with ethanol are located on the positive end of function 2 and the coefficients with higher positive influence in these functions are *m*/*z* 45, *m*/*z* 137, and *m*/*z* 163. On the contrary, KER samples are those that show highest negative values in function 2. *m*/*z* 72, *m*/*z* 94, and *m*/*z* 130 are the signals with highest negative coefficients in this function. The samples can be discriminated with regards to both functions.

**Figure 5:**
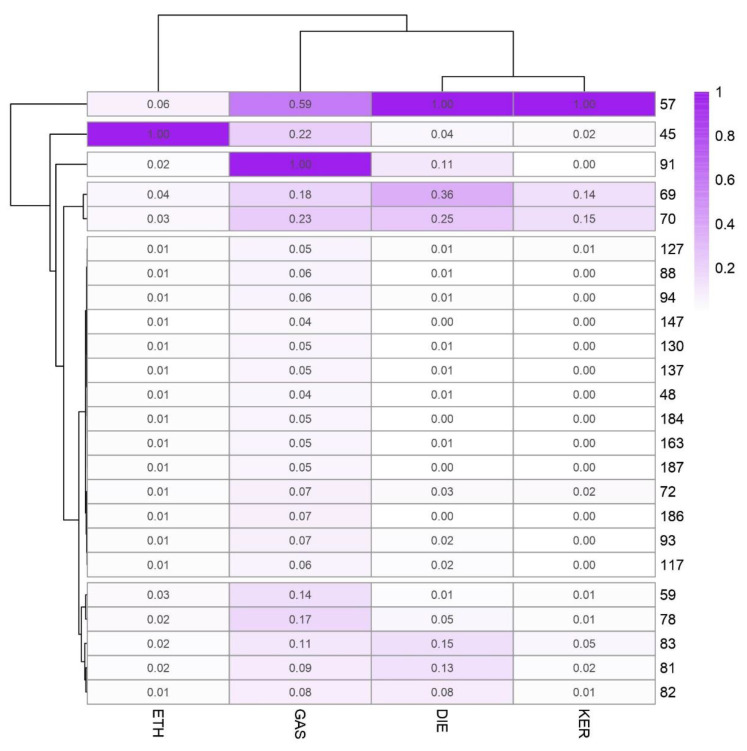
Intensity values of the selected *m*/*z* in the LDA for the discrimination of ILRs.

Figure 5 represents the average values at the different *m*/*z* selected from LDA for each ILR in a heat map plot. All the *m*/*z* values were normalized to the base peak at 100%. For a better understanding, a cluster is displayed to group the samples and the variables. For this cluster, Ward´s method with Manhattan distances was used. As Figure 5 shows, there is a noticeable difference between groups of ILR samples. Samples burned with gasoline have the highest intensity with *m*/*z* 91 and a high positive value from discriminant function 1.

According to the major ions in mass spectra of common ILs included in ASTM E1618 [9]. *m*/*z* 91 is characteristic for C-2, C-3, and C-4 alkylbenzenes commonly abundant in GAS samples. *m*/*z* 57 is related to alkanes, commonly present in heavier petroleum distillates. For this reason, is the most important signal in terms of abundance in DIE and KER samples, but also it is found at a lower level in GAS and ETH. *m*/*z* 69, *m*/*z* 70 together with *m*/*z* 57 are compounds typically found in heavy petroleum distillates. Whilst *m*/*z* 69 is related to both cycloalkanes and alkenes, *m*/*z* 57 is associated to alkanes. As the results suggest, *m*/*z* 81, *m*/*z* 82, and *m*/*z* 83 let us discriminate between DIE and KER. *m*/*z* 82 and *m*/*z* 83 are both related to *n*-alkylcyclohexanes. The samples burned with ethanol present only one characteristic signal at *m*/*z* 45. This intensity is related to alcohols.

## 4. Conclusions

HS-MS eNose combined with HCA and LDA was used to develop and validate a robust method for the discrimination of different ILRs (gasoline, diesel, charcoal starter with kerosene, and ethanol) in fire debris samples that contained interfering background compounds (petroleum-based substrates). Additionally, samples were taken after different sampling times in order to reproduce closer to actual fire investigation conditions. HCA results showed a strong tendency of the fire debris samples to be grouped based on the substrate used and the type of IL. This suggested that petroleum-derived substrates have common signals with ILs, making it difficult to classify them correctly by unsupervised technique. Nevertheless, by applying LDA on the TIS, a full discrimination of all the samples with and without ILR was achieved regardless the substrate or the sampling time. Based on these results, the proposed method allows the detection of ILR in fire debris even when complex matrices are burned. An additional LDA allowed for the complete discrimination between the four ILRs. However, samples burned with gasoline or ethanol formed more heterogeneous groups, and therefore, sampling time or substrates may affect the analyses of these ILRs. Although gasoline and ethanol ILRs were actually discriminated, based on these results, careful analysis are to be carried out when these types of substrates are found at the fire scene. In short, these results also demonstrate how static headspace directly injected in a mass spectrometer without any chromatographic separation in combination with suitable chemometric tools can be used for the identification of fire debris ILRs. Specifically, HS-MS eNose combined with LDA can be a solid help for forensic analysts to interpret analyses’ results with a more rapid, systematic and, most important, objective method. 

## Figures and Tables

**Table 1:** Fisher´s linear discrimination functions for fire debris samples burned with/without IL obtained from the linear discriminant analysis (LDA).

Classification Function Coefficients					
*m*/*z*	Fire Debris		*m*/*z*	Fire Debris	
	without IL	with IL		without IL	with IL
45	25.549	139.818	104	–19.066	–276.990
46	11.554	79.221	105	–15.516	25.072
53	40.396	–180.484	114	2.931	–259.340
57	28.633	−2.003	136	–29.525	99.590
59	–36.423	–155.993	137	–5.907	250.730
60	–6.324	–284.772	141	13.245	–212.961
64	25.736	–331.332	142	–74.702	152.084
65	43.271	235.297	143	–34.091	271.643
71	–11.225	459.551	156	–11.293	210.897
72	45.816	–279.873	160	28.248	–252.297
77	24.696	109.341	169	–49.213	56.574
89	88.155	–265.753	181	9.034	337.532
91	0.834	73.420	193	49.974	–206.138
93	–65.970	–184.987	197	–53.574	259.863
97	–1.042	111.749	(Constant)	–18.783	–78.320
